# Roles of Immune Cells in Hereditary Angioedema

**DOI:** 10.1007/s12016-021-08842-9

**Published:** 2021-05-29

**Authors:** Anne Lise Ferrara, Leonardo Cristinziano, Angelica Petraroli, Maria Bova, Maria Celeste Gigliotti, Simone Marcella, Luca Modestino, Gilda Varricchi, Mariantonia Braile, Maria Rosaria Galdiero, Giuseppe Spadaro, Stefania Loffredo

**Affiliations:** 1 Department of Translational Medical Sciences, University of Naples Federico II, 80131 Naples, Italy; 2 Center for Basic and Clinical Immunology Research (CISI), University of Naples Federico II, Naples, Italy; 3 WAO Center of Excellence, Naples, Italy; 4 Institute of Experimental Endocrinology and Oncology (IEOS), National Research Council, Naples, Italy

**Keywords:** Angiogenesis, Endothelial cell, Lymphocyte, Macrophage, Mast cell, Monocyte, Neutrophil

## Abstract

Hereditary angioedema (HAE) is a rare genetic disease, characterized by recurrent and unexpected potentially life-threatening mucosal swelling. HAE may be further classified into HAE with C1‐inhibitor deficiency (C1‐INH‐HAE) and HAE with normal C1‐INH activity (nlC1‐INH‐HAE), mostly due to mutations leading to increased vascular permeability. Recent evidence implicates also the innate and adaptive immune responses in several aspects of angioedema pathophysiology. Monocytes/macrophages, granulocytes, lymphocytes, and mast cells contribute directly or indirectly to the pathophysiology of angioedema. Immune cells are a source of vasoactive mediators, including bradykinin, histamine, complement components, or vasoactive mediators, whose concentrations or activities are altered in both attacks and remissions of HAE. In turn, through the expression of various receptors, these cells are also activated by a plethora of molecules. Thereby, activated immune cells are the source of molecules in the context of HAE, and on the other hand, increased levels of certain mediators can, in turn, activate immune cells through the engagement of specific surface receptors and contribute to vascular endothelial processes that lead to hyperpemeability and tissue edema. In this review, we summarize recent developments in the putative involvement of the innate and adaptive immune system of angioedema.

## Introduction

Angioedema is a self‐limiting tissue swelling due to periodic increase in vascular permeability caused by the release of bradykinin (BK) and/or other cell–derived mediators. Recurrent swellings are localized to the skin and/or to the upper respiratory, gastrointestinal, and genitourinary tracts [1]. Angioedema can be hereditary or acquired. The most common form of hereditary angioedema (HAE) is caused by deficiency of C1 esterase inhibitor (C1‐INH‐HAE), but HAE can also occur with normal levels of C1‐INH (nl‐C1‐INH‐HAE) [1].

C1-INH is a protein of the complement system which is a critical component of both the innate and adaptive immunity [2–4]. The immune system is typically divided in two branches: innate and adaptive, although these distinctions are not completely exclusive [5]. The fundamentals of HAE (or angioedema) have been extensively reviewed previously [1, 6]. In this paper, we focus our discussion on the roles played by the immune system in the pathophysiology of angioedema.

## Innate Immune System

### Monocytes

Monocytes (MO) originate from myeloid progenitors in the bone marrow (BM). These cells are rapidly recruited to tissues during infections and inflammation, where they differentiate into macrophages or dendritic cells [7]. There are three subsets of human MO: classical (~90%), intermediate, and non-classical (~10%) [8]. These subpopulations can be further characterized by different functions and expression of surface markers and chemokine receptors [9]. They display phagocytic and microbial activity and produce pro-inflammatory cytokines. Intravital microscopy studies have revealed that non-classical MO continuously monitor the vasculature under physiological conditions through an LFA/ICAM-dependent crawling mechanism on resting endothelial cells (EC) [10, 11]. The role of MO has been poorly studied in HAE. It would be interesting to evaluate whether the surveillance of EC integrity driven by non-classical MO is altered in HAE patients which have an abnormal basal vascular permeability [12].

**Fig. 1:**
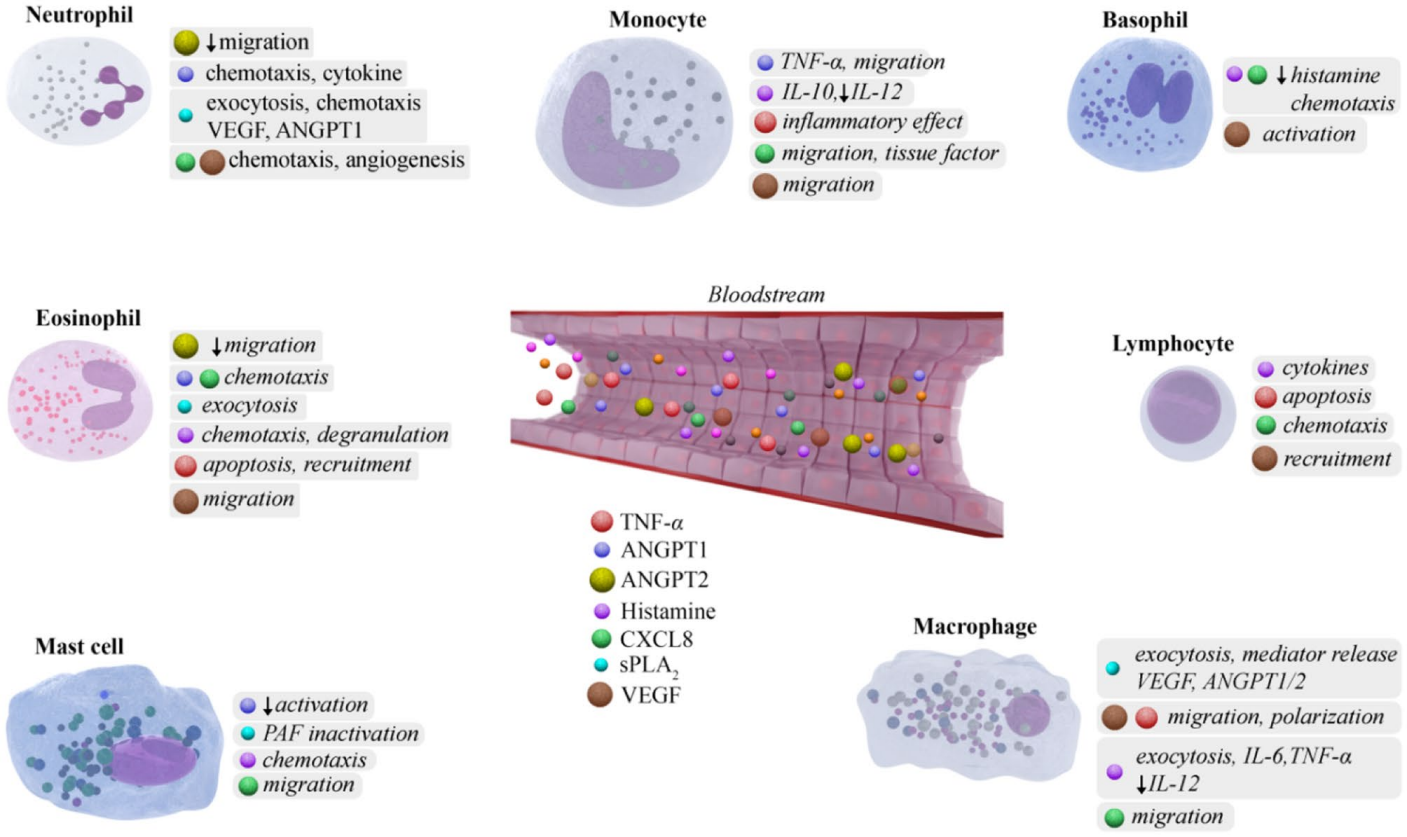
Schematic representation of the effects of mediators increased in C1-INH-HAE on different immune cells

As an example, endothelial permeability is mediated by vasoactive mediator release, including vascular endothelial growth factors (VEGFs) [13] that were found increased in C1-INH-HAE patients and correlate with disease severity [14, 15]. VEGFs are produced by various cells including EC. They signal through the tyrosine kinase receptors, VEGFR‐1, VEGFR‐2, and VEGFR‐3 [16]. Indeed, MO express low levels of VEGFR‐1 and VEGFR-3 but do not express VEGFR‐2. MO produce high amounts of VEGF (in response to M‐CSF) or the antagonistic soluble VEGFR‐1 (in response to GM‐CSF) [8]. Increased VEGF-A in C1-INH-HAE could be caused by MO activation or vice versa circulating VEGF through binding to VEGFR-1 could attract and activate circulating MO (Table 1; Fig. 1).

**Table 1:** Mediators involved in HAE and their cellular sources

Mediators	Concentration in HAE		Cellular source	References
	During remission*	During attack**		
Adrenomedullin	Unchanged	Increased	MO	[135, 147]
ANGPT1	Increased	Increased	Baso, MA, MC, PMN	[14, 41, 46, 47, 148]
ANGPT2	Increased	Unchanged	Baso, MA, MC	[14, 41, 47, 148]
CXCL8	Unchanged	Increased	Baso, MA, MC, MO, PMN	[41, 63, 97, 149–151]
Elastase	Unchanged	Increased	MC, PMN	[63, 152]
Histamine	Increased	Unknown	Baso, MC	[109], this article
Myeloperoxidase	Unchanged	Increased	PMN	[63, 153]
PAF-AH	Increased	Reduced	MC	[114, 154]
Pentraxin	Unchanged	Increased	MA, PMN	[155, 156]
ROS	Increased	Unknown	MA, MC, MO, PMN	[57, 157–159]
sPLA_2_	Increased	Reduced	Eos, MC, PMN, T cell	[46, 80, 114, 160, 161]
Tissue factor	Increased	Unchanged	MO	[29]
TNF-*α*	Decreased	Increase	MA, MC, MO,	[162–164]
Tryptase	Unchanged	Unknown	Baso, MC	[109] this article,
VCAM-1	Increased	Unknown	MA	[165, 166]
VEGF	Increased	Unchanged	Baso, MA, MC, MO, PMN	[14, 41, 47, 80, 95, 167]

*Baso* basophil, *Eos* eosinophil, *MA* macrophage, *MC* mast cell, *MO* monocyte, *PMN* neutrophil

*Compared with healthy controls; **Compared with remission phase

Additionally, MO secrete a wide spectrum of mediators including complement components such as C1-INH [17]. Hepatocytes are the major cellular source of plasma C1-INH [18]. MO contribute to C1-INH production, particularly at the site of inflammation where INF-*γ* is a potent inducer [19, 20]. Understanding the regulation of C1-INH synthesis by MO is essential to evaluate their potential role in C1-INH-HAE.

Lipopolysaccharide (LPS) does not trigger an increase in C1-INH levels in MO cultures, but it induces high levels of IL-1. LPS may play a role in regulation of C1-INH synthesis through the induction of IL-1, which is essential for T cell activation to yield IFN-*γ* through the induction of C1-INH in MO and hepatocytes [21]. LPS raised also C3 production by MO [22] but did not stimulate C1q and C1s secretion. Moreover, C2 was increased by IFN-*γ* to a similar extent as C1-INH [23, 24], whereas it did not affect C3 synthesis [23]. MO do not produce C4 in MO culture supernatants [17].

**Fig. 2:**
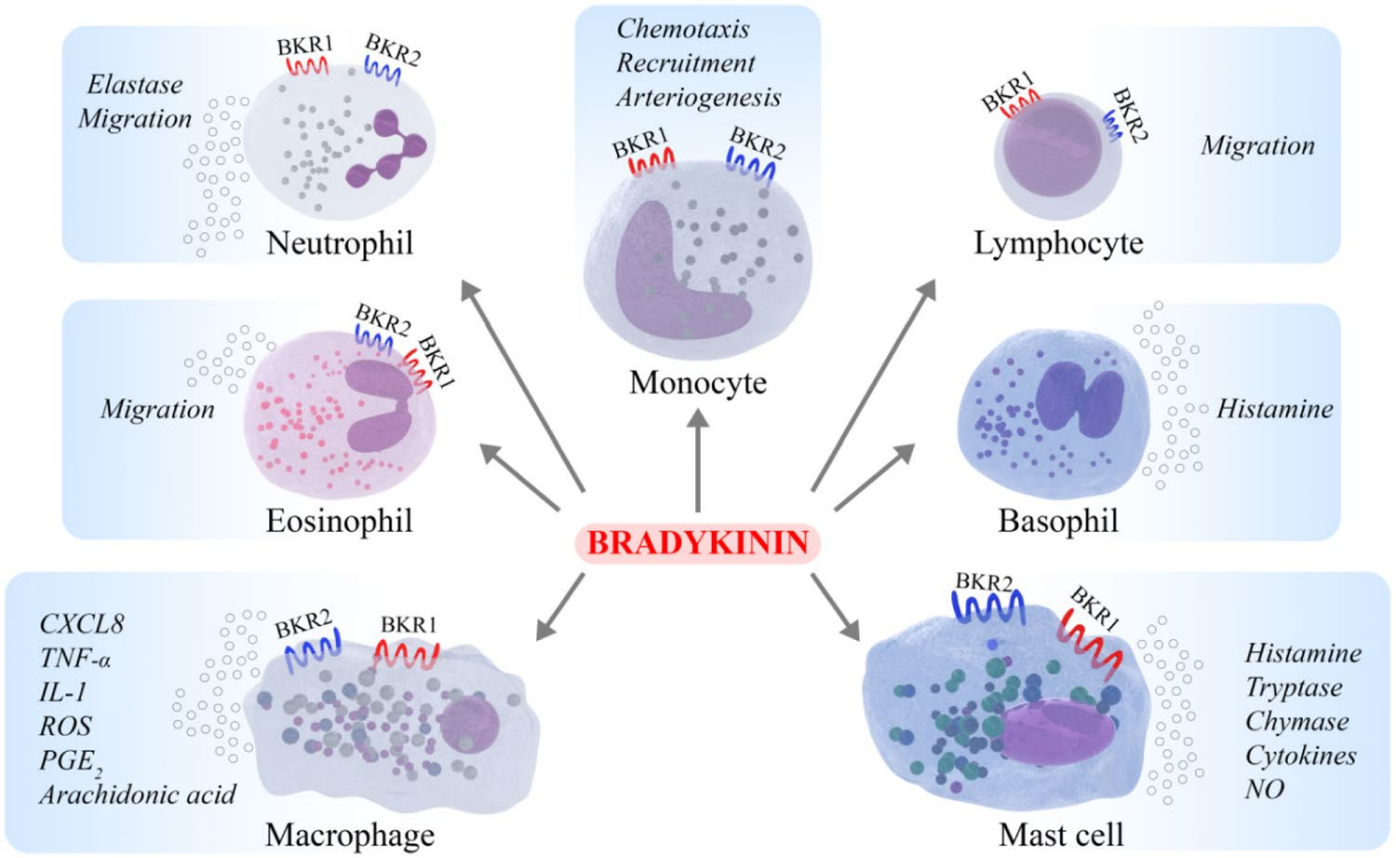
Schematic representation of bradykinin effects on different immune cells

Another link to the pathophysiology of C1-INH-HAE might be the effect of BK on MO; BK is formed downstream the kallikrein-kinin system (KKS) and is unquestionably the most important mediator in C1-INH-HAE [25]. The vasoactive effects of BK are mediated by the cell surface BKR1 and BKR2 receptors expressed on several cell types. The existence and modulation of these receptors in MO are still limited. Activation of BKR1 promotes MO chemotaxis and arteriogenesis, whereas BKR2 signaling governs MO recruitment (Fig. 2) [26–28]. Therefore, it can be hypothesized that the BK increase in HAE could cause an activation of circulating MO.

Expression of tissue factor (TF) by MO may represent another link between these cells and angioedema. TF may play a role in angioedema attacks by activating the coagulation pathway in association with reduced functions of C1‐INH [29]. TF, predominantly expressed in vessel wall, forms a complex with FVIIa and initiates the extrinsic coagulation pathway. TF is also present in the cytoplasm and on the surface of MO and can be released by various exogenous/endogenous inflammatory stimuli. The TF expression is up-regulated by LPS, immune complexes, C5a, cytokines, and oral contraceptives, which have been suggested as risk factor for angioedema [29]. TF is expressed in urticarial skin lesions with evidence of activation of the extrinsic pathway [30]. An increase of TF was demonstrated on MO of patients with C1-INH-HAE during remission compared with healthy controls [29]. C1‐INH efficiently inhibited LPS‐induced TF on MO, suggesting that MO of HAE due to C1‐INH deficiency are susceptible to induction TF expression. This is supported by four‐fold increase in TF expression a day after the infusion of C1‐INH [31]. However, no difference in TF expression was shown between remission and attack of HAE [31]. Taken together, in HAE the combination of low C1‐INH activity, subclinical activation of extrinsic coagulation pathways triggered by TF, may be important to the pathophysiology of angioedema. Further studies are needed to confirm whether the increase of TF expression on MO and the coagulation system activation observed is the cause or just an epiphenomenon in HAE attacks [29]. The role of MO in the pathophysiology of angioedema involves several mediators that can modulate MO activation (Table 1; Figs. 1 and 2).

### Macrophages

The barrier properties of EC are critical for the maintenance of fluid and protein balance between the intravascular and extravascular compartments [32]. Imbalance of these barriers is implicated in the genesis or progression of angioedema [12]. The altered barrier function is linked to the release of a variety of soluble mediators acting on EC [32, 33] produced by resident cells, including macrophages [34].

Macrophages (MA) are innate immune cells that are localized in various tissues [35]. Most MA are derived from MO that migrate into connective tissues [36]. MA exert a variety of functions like phagocyting foreign agents, digesting dead cells, and regulation of innate immune response by releasing of several molecules [37]. Mediator secretion by MA is implicated in several disease states ranging, from chronic inflammation to allergy [36]. MA release upon inflammatory stimulation a plethora of inflammatory (e.g., TNF-*α*, IL-1, IL-6, CXCL8, IL-12) and anti-inflammatory cytokines (IL-10 and TGF-*β*) [36]. Several MA-derived cytokines are altered in serum of HAE patients [38] (Table 1). Concentrations of IL-1, IL-6, and TGF-*β* are significantly higher in HAE patients in remission compared with healthy controls [38]. These cytokines, plus IL-10, are further increased in HAE patients during attacks compared with asymptomatic period. In addition, IL-1 and TNF-*α* have been reported to stimulate EC and augment activation of the prekallikrein (PK)–high molecular weight kininogen (HK) complex, suggesting a possible role in the pathophysiology of HAE [39].

MA are a major source of VEGFs and angiopoietins (ANGPTs) [40, 41]. Their concentrations are increased in C1-INH-HAE patients and correlate with disease severity [14, 15] (Table 1).

Extracellular or secreted phospholipases A_2_ (sPLA_2_s) also modulate EC and vascular permeability. PLA_2_s enzymes hydrolyze membrane glycerol-phospholipids to release arachidonic acid and lyso-phospholipid [42, 43]. sPLA_2_s can modulate vascular permeability either by directly activating EC or by catalyzing the production/degradation of vasoactive molecules [43]. MA are a target for sPLA_2_ (Fig. 1). These enzymes activate MA and induce the production of cytokines, chemokines, VEGFs, and ANGPTs [40, 44, 45]. We found that sPLA_2_ activity is increased in biological fluid of C1-INH-HAE patients during symptom-free period compared with healthy controls [46]. sPLA_2_ group IIA (PLA2G2A) in C1-INH-HAE increases endothelial permeability and impairs C1-INH functional activity in vitro [46]. ANGPT1, a unique vascular stabilizer, is further increased during angioedema attack, whereas sPLA_2_ activity is decreased [46, 47] (Table 1).

Since BK is a potent vasodilator, promoter of vascular permeability [25]. It hypothesized that activated BKR2 on EC and/or on MA and/or mast cells may account for the altered levels of cytokines, angiogenic/lymphangiogenic factors, and sPLA_2_ in C1-INH-HAE patients [16, 48, 49]. MA express both BKR1 and BKR2 [50]. BK also induces TNF-*α* and IL-1 release from murine MA cell lines [52, 53] and stimulates prostaglandin E_2_ production from rat peritoneal MA [54] (Fig. 2). The activation of BKRs increases intracellular free calcium, which activates sPLA_2_ and consequently induces arachidonic acid release and its metabolites [51]. BK is more potent to activate intermediate-size MA compared with smaller peritoneal and alveolar MA [50]. Collectively, these findings indicate that overproduction of BK in HAE patients may affect the MA activation and their inflammatory responses in vivo.

MA are well known for their phagocytic activity and are highly specialized in removal of dying or dead cells [37]. Phagocytosis is facilitated by opsonization, a process by which serum components tag pathogens for recognition by MA and neutrophils. Opsonization is mediated by C1, C3, and C4 which are components of the *c*omplement classical pathway [3].

In C1-INH-HAE patients, low concentration/activity of C1-INH causes a gradual consumption of complement proteins in serum. Sera from HEA patients reduced the ability of MA to phagocyte apoptotic cells compared with sera from healthy donors [55]. Therefore, C1-INH-HAE patients can have immunological abnormalities due to decreased levels of complement components, which give rise to a lower capacity for opsonization from phagocyte cells including MA [55].

In conclusion, the role of MA in the pathophysiology of HAE is not yet completely understood. However, the ability of MA (1) to modulate vascular permeability by catalyzing the production of vasoactive molecules; (2) to be activated by key mediators of HAE, such as BK and sPLA_2_; and (3) to modify opsonization capacity suggests that these cells play an important role in both asymptomatic and symptomatic phases of HAE.

### Neutrophils

Neutrophils, or polymorphonuclear leukocytes (PMN), are major effectors in innate immunity and acute inflammation [56]. They are circulating cells that must be lured into inflamed tissue by crossing the endothelial barrier. Sequential adhesive interactions between PMN and ECs are required for PMN extravasation. Adhesion molecules (i.e., ICAM-1, VCAM) lead to adhesion and arrest onto the endothelium and a subsequent PMN transmigration in the tissue where they play a critical role in pathogen elimination and tissue repair by releasing several cytotoxic products and reactive oxygen species (ROS) [57]. New evidences have highlighted our knowledge on PMN as cells playing a role beyond the acute infection including HAE [58].

PMN count is increased in C1-INH-HAE patients during edematous episodes. This PMN imbalance was attributed to the hemoconcentration caused by plasma extravasation during angioedema attack [59–62]. Veszeli et al. demonstrated a higher PMN count also in C1-INH-HAE patients during symptom-free period compared with healthy controls [63]. These authors described an increased release of neutrophil granule-derived enzymes in plasma (i.e., myeloperoxidase (MPO), elastase (NE), and pentraxin 3 (PTX3)) during attacks but not during attack-free period and in healthy controls. Plasma concentration of these enzymes was correlated with neutrophil counts. The increased plasma levels of MPO, NE, and PTX3 were attributed to neutrophil extracellular trap (NETs) release. Interestingly, CXCL8 and TNF-*α* levels, both involved in PMN activation and/or released by neutrophils, were also altered in C1-INH-HAE patients during acute phase compared with symptom-free period [63] (Table 1). Grymova and colleagues confirmed PMN activation and dysregulation in C1-INH-HAE type I and II patients [64]. mRNA expression of 10 genes related to PMN activation (CD274, IL1*β*, IL1RN, CXCL8, MMP9, and TLR4) was increased in HAE patients in symptom-free periods compared with healthy donors in addition to increased CD11b, decreased CD16 plasma membrane deposition, and increased relative CD274^+^ and CD87^+^ neutrophil counts, but not by increased NE or MPO plasma levels (Table 1). In addition, a co-culture of PMN and T-cells/PBMC showed a suppressive function of patient’ PMN resulted from a decreased CD25^+^ and IFN-*γ*^+^ T-cell/PBMC ratio in patients [64].

PMN can interact with the contact system in order to boost neutrophil extravasation induced by BK-mediated vasodilatation [65]. Brower et al*.* reported that NE can inactivate C1-INH allowing to contact system activation [66]. In addition, in vitro studies showed that BKR1 on ECs regulates neutrophil trafficking [67, 68]. BK levels are increased in HAE patients compared with healthy controls leading to PMN activation. In fact, BK increased the PMN adhesion to ECs [69, 70] and induced only a moderate migration of human peripheral PMN in BKR2-manner [51]. In addition, BK mediated NE release by PMN [71] (Fig. 2).

Neutrophil-derived proteinase 3 can proteolyze HK and liberate proteinase 3-kinin, thereby initiating kallikrein-independent activation of the kinin pathway [72]. Wachtfogel et al*.* reported that kallikrein (PKa) and Factor XIIa (FXIIa) can induce PMN degranulation [73]. Finally, it has been shown that NETs can activate FXII through several mechanisms. The negative charge of DNA could contribute to auto-activation of FXII or can sequester FXII and present it for activating cleavage [74].

PMN are source and/or target of several mediators and play a role in different context (i.e., inflammation, angiogenesis) [75]. Different isoforms of sPLA_2_s (e.g., groups II, V, and X) can activate human PMN inducing NE, CXCL8, or angiogenic factors (VEGFs and ANGPTs) release [76–80]. Therefore, increase of PLA2G2A in C1-INH-HAE patients could affect the activation of PMN and be responsible of release for the VEGFs and ANGPTs [14, 46, 47] (Fig. 1). Taken together, these results confirm the involvement of PMNs in the pathophysiology of angioedema by releasing mediators and induce endothelial preconditioning state, thereby predisposing HAE patients to edema formation.

### Eosinophils and Basophils

Eosinophils and basophils are immune cells activated in several pathological conditions (e.g., allergic diseases, infections, cancer) [81, 82]. These cells are characterized by different phenotypes and by the ability to respond to specific stimuli, activating and inhibiting surface receptors [83]. Eosinophils derive from CD34^+^CD117^+^ hematopoietic stem cells in the BM. After their maturation they enter the circulation [84]. Activated eosinophils release pro-inflammatory cationic proteins, cytokines and chemokines, angiogenic [49, 85], and lipid mediators [86]. They also migrate to the inflammatory sites through the adhesion to activated ECs [84, 87, 88]. The involvement of eosinophils in HAE has not been investigated. They could be the source of VEGFs, TNF-*α*, and sPLA_2_ found in plasma of HAE patients. Moreover, eosinophils can be activated by BK leading to elastase release [71] and chemotaxis [89] (Fig. 2).

Episodic angioedema with eosinophilia (EAE), also known as Gleich syndrome, is a rare disorder characterized by recurrent episodes of angioedema, urticaria, fever, and marked eosinophilia that occur at 3–4 week intervals [90]. A peak of marked eosinophilia is preceded by a rise in serum IL-5 and IL-13 in EAE patients [91]. These findings suggest that immune cell-derived Th2-like cytokines are involved in this form of angioedema. However, the etiology of the cycling angioedema and eosinophilia remains to be elucidated.

Basophils represent less than 1% of peripheral blood leukocytes, and their activation leads to histamine release [92]. They are rarely present in tissues unless inflammation occurs [93, 94]. Basophil activation induces the release of VEGF-A [95, 96] and ANGPT1 and ANGPT2 (Table 1). Human basophils also produce CXCL8 [97]. Some of those mediators (e.g., VEGFs, ANGPTs, sPLA_2_, and CXCL8) were altered in HAE contributing to alteration of vascular homeostasis. Collectively, these data may suggest a potential role of eosinophils and basophils in the pathophysiology of certain forms of HAE.

### Mast Cells

Mast cells (MC) can be identified in blood vessels, within mucosal and epithelial tissues and in the terminal nerve endings [98]. These cells release several preformed pro-inflammatory mediators (e.g., histamine, tryptase, chymase) [99]. MC derive from CD34^+^CD117^+^ (KIT) hematopoietic stem cells in the BM [100] and migrate as immature progenitor cells through the bloodstream to peripheral tissues where they complete maturation [101].

In this paper, we discuss the role of MC in angioedema without wheals. MC-mediated angioedema is pathophysiologically similar to urticaria, although it occurs in deeper levels of the dermis and involves probably different mediators. Except for C1-INH-HAE, the pathophysiology of angioedema without wheals is not completely clear. MC release several vasoactive mediators (e.g., histamine, prostaglandins, cysteinyl leukotrienes) contributing to extravasation of fluid in the deeper layers of the skin/mucosa of angioedema patients [102]. The canonical mechanism of MC activation is IgE-mediated [101]. However, in most patients, angioedema develops without an interaction between IgE-antigen complex bound to MC. Several non-IgE-mediated stimuli (e.g., drugs, C5a, C3a) can induce human MC degranulation [103].

**Fig. 3:**
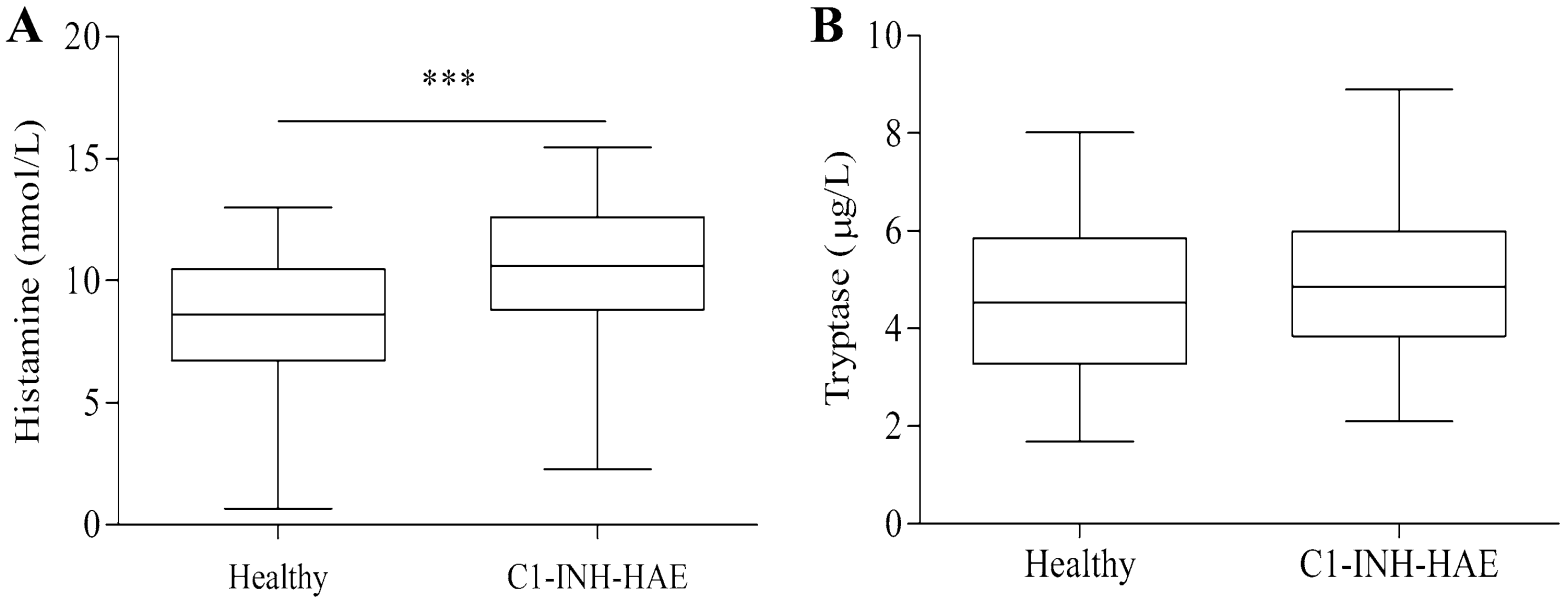
Plasma concentrations of histamine and tryptase in C1-INH-HAE patients. The graph depicts plasma **histamine** (**a**) and **tryptase** (**b**) in 64 controls (Healthy) and in 102 patients with C1-INH-HAE in remission. Histamine was measured by ELISA. Tryptase was measured by fluoro-enzyme immune assay using Uni-CAP100. Data are shown as the median (horizontal black line), the 25th and 75th percentiles (boxes) and the 5th and 95th percentiles (whiskers) of 64 controls and 102 patients

Histamine is a relevant vasoactive amine contained in MC granules. It binds to H1-receptors on ECs, inducing vasodilatation; increases blood flow; and causes vessel [104]. Histamine stimulates nitric oxide expression and increases blood flow and plasma extravasation causing angioedema [105]. Most cases of angioedema are attributable to the vasoactive mediator BK and histamine. MC express BKR2 through which BK induces histamine release [106–108] (Fig. 2). Angioedema attacks of HAE patients are unresponsive to antihistamines and glucocorticoids [109]. Histamine is presumably not the main mediator of angioedema. In this paper, we present original results indicating that 102 patients with C1-INH-HAE in remission have increased concentrations of histamine compared with 64 healthy controls (Fig. 3a). We also measured the tryptase concentrations in these patients. Tryptase is a specific marker of MC activation [110]. We found that tryptase levels in C1-INH-HAE patients in remission are not altered compared with controls (Fig. 3b). These results are of some interest because histamine is secreted by basophils and MC, whereas tryptase is essentially released by MC [92, 111]. Therefore, it is possible to hypothesize that the increase in blood histamine in patients with C1-INH-HAE in remission derives essentially from basophils rather than MC. It would be interesting to evaluate tryptase and histamine levels during attacks in order to better understand their roles and the cellular sources (MC and/or basophils) in acute phase of angioedema.

Tryptase releases BK mainly through plasma kallikrein (PKa) activation and enhanced vascular permeability [112]. Sala-Cunill et al*.* demonstrated that tryptase levels are correlated to plasma HK cleavage during anaphylaxis [113]. These findings could indicate that tryptase might contribute to kinin cleavage and consequently BK production in angioedema.

Stimulation of MC can release PLA_2_. Human lung MC express and release multiple sPLA_2_s when activated by anti-IgE [114]. The sPLA_2_s released by MC contribute to leukotriene C_4_ production by acting in an autocrine fashion. PLA_2_ plasma activity was increased in patients with C1-INH-HAE during remission and was decreased during acute attack perhaps because of their activation and internalization in EC [46]. The cellular sources of these mediators remain unclear but could be partially attributable to MC activation in C1-INH-HAE (Table 1).

The proteoglycan matrix in MC cytoplasmic granules is mostly composed by heparin [115]. Heparin can initiate in vivo the contact system cascade activating FXIIa [116] and in turn BK production. Heparin appears to have a dual function in FXII activation: it has the negatively charged surface for binding and activation of plasmatic FXII [117]. and it blocks FXII inhibition binding antithrombin III [118]. Oschatz et al*.* described a paracrine mechanism by which MC-released heparin induces contact system-driven edema in mice [119]. These results suggest that MC activation and heparin can contribute to attacks in HAE patients [119].

Another MC mediator potentially capable of activating the contact system is elastase. It cleaves the light chain of HK and seems to be a positive regulator of the contact system activation [120]. Taken together, these findings indicate that MC degranulation may trigger FXII activation and the generation of BK through the release of heparin, tryptase, and elastase or other mediators. The potential roles of other MC mediators in contact system activation need to be further explored.

## Adaptive Immune System

### Lymphocytes

Lymphocytes mediate adaptive immune responses by providing the lifelong immunity following exposure to antigens [4]. These cells originate in the BM and migrate to tissues by circulating in the blood and in the lymphatic system [121]. There are several different lymphocytes including B and T cells, natural killer cells (NK cells), and innate lymphoid cells [122, 123]. Activated B lymphocytes differentiate into plasma cells, which secrete antibodies. T cells include by two main classes: CD8^+^ cytotoxic T cells and CD4^+^ T cells [124]. T and B cells express different receptors to recognize a wide spectrum of antigens [124]. The antigen receptor of B lymphocytes is the membrane isoform of IgM [125]. The T cell receptor (TCR) on T cells recognizes protein antigens or metabolized by dendritic cells and MAs.

Adaptive immunity has been poorly studied in HAE. Few reports have reported abnormal T and B cell counts, abnormal distribution of T cell surface IgG-receptors, and reduced Langerhans cell numbers in HAE patients [126, 127].

Several studies examined the involvement of cytokines in HAE. Arcoleo et al*.* comparing C1-INH-HAE patients before and after the acute attack with matched control subjects observed several modifications of IL-17 lymphokine network [38]. IL-17 concentrations were increased, whereas IL-23 levels were unmodified and TGF-*β*3 concentrations were reduced [38]. Comparing healthy and HAE subjects in remission, they found a significant difference for IL-17, GM-CSF, IL-21, and TGF-*β*1/2 [38]. These suggests that in HAE subjects there is a cytokine milieu favoring expansion of Th17 or Th17-type subsets capable of producing cytokines associated with contact activation by BK leading to local angioedema formation responsible of increase in permeability and subcutaneous swelling [38, 128, 129]. Th17 expansion could down-modulate inflammatory response favoring the natural resolution of angioedema [38, 130–133].

The alterations of circulating cytokines suggest HAE is a complex disorder caused by generation of BK associated with increase in several cytokines (Fig. 3). Lopez-Lera et al*.* evaluated the expression of HAE by profiling the RNA expression of peripheral blood mononuclear cells (PBMC) from C1-INH-HAE families [134]. This study did not reveal alterations in the expression pattern of PBMC in association to frequency and severity of disease [134]. Castellano et al., using a different approach, explored the involvement of several putative genes by performing a microarray gene expression analysis on RNA isolated from PBMC of HAE patients during attacks and in remission. They demonstrated the up-regulation of adrenomedullin (ADM) and cellular receptor for urokinase plasminogen activator (uPAR), during the acute attack. These gene activations involved in vascular tone regulation and in inflammatory response might have a pathogenic role by amplifying BK production and edema formation in HAE patients [135]. uPAR is a glycosylphosphatidylinositol-anchored protein [136] that binds uPA [137]. The function of uPA is the conversion of plasminogen to plasmin. uPAR is expressed by resting granulocytes and monocytes and by activated lymphocytes [138]. uPAR interacts with components of the BK-forming cascade. The neutralization of uPAR expressed on T cells leads to a reduction of BK. This observation highlights a potential role for adaptive immunity to modulate the edema formation through regulation of BK production [135].

Patients with HAE tend to produce autoantibodies. Kessel et al. demonstrated that HAE patients have an increase of autoantibodies presumably due to the activation of B cells associated with over-expression of TLR9 which plays a role in the induction and maintenance of autoimmunity [139].

### Infection/Inflammation

As previously mentioned, an interesting aspect is the possibility that infections trigger angioedema attacks [140]. Bacteriuria and Helicobacter pylori could represent triggers of angioedema attacks [141, 142]. The observations were attributed to the excessive consumption of complement by antibodies directed against bacteria. The antibody response and the formation of immune complexes may trigger the consumption of already reduced C1-INH in HAE patients [134, 143].

Neutrophil-lymphocyte ratio (NLR) is a simple and easily used parameter for the assessment of inflammation. It has been found a positive correlation between the angioedema attack and NLR [144] suggesting that the NLR could be useful as a predictive biomarker for prediction of the attack in HAE patients.

To conclude this section, the roles of different subsets of lymphocytes in the pathophysiology of angioedema have not been thoroughly studied. Cells of adaptive immunity could have a role in the regulation of the severity of this disease in different forms of angioedema. Further studies with RNA sequencing and proteomic technologies will clarify the possible roles of multiple cells involved in adaptative immunity in the pathophysiology of angioedema attack.

## Conclusions

In this review, we have summarized the results of relatively few studies examining the roles played by immune cells presumably involved in HAE. We have tried to distill the contribution that each immune cell can exert directly or indirectly in the pathophysiology of angioedema. The genetics and the resulting protein alterations of the majority of HAE patients are well characterized.

Acute phase of a disease is characterized by a transient increase of vascular permeability followed by the formation of local edema. Current research is focusing on EC receptors and the mechanisms of their activation in different phenotypes of angioedema. There is compelling evidence that the endothelium actively participates in both innate and adaptive immune responses. EC are in a strategic location to activate the circulating immune cells and those that transmigrate across the endothelium into the tissues. The roles played by EC in the recruitment of immune cells into lymph nodes and tissues highlight an intimate relationship between EC and immune cells [145, 146]. Therefore, the altered vascular permeability in both remission and acute phase of HAE can affect the effector functions of several immune cells.

The circulating levels of several mediators are altered in remission and/or during attack in HAE patients (Table 1). Activated immune cells might be the source of these molecules in the context of HAE. Moreover, the increased levels of several mediators can, in turn, activate the immune cells through the engagement of specific surface receptors (Fig. 1). In this paper, we have also discussed the effects of a variety of mediators on immune cells (Fig. 2)_._

In summary, it appears that the role of the multiple cells of innate and adaptive immune system in the pathophysiology of angioedema has not been thoroughly investigated. A better knowledge of these mechanisms could open new diagnostic and therapeutic opportunities for the different forms of angioedema.

## Abbreviations

AAE: Acquired angioedema
ADM: Adrenomedullin
ANGPT: Angiopoietin
b-FGF: Basic fibroblast growth factor
BKR: Bradykinin receptor
BK: Bradykinin
C1-INH: C1-inhibitor
EAE: Episodic angioedema with eosinophilia
EC: Endothelial cell
ECP: Eosinophil cationic protein
EDN: Eosinophil-derived neurotoxin
EPX: Eosinophil peroxidase
fMLP: N-formyl-methionine-leucyl-phenylalanine
FXII: Factor XII
GM‐CSF: Granulocyte–macrophage colony‐stimulating factor
HAE: Hereditary angioedema
His: Histamine
HK: High molecular weight kininogen
ICAM: Intercellular adhesion molecule
INF: Interferon
KKS: Kallikrein-kinin system
LFA: Lymphocyte function-associated antigen
LPS: Lipopolysaccharide
MA: Macrophage
MBP: Major basic protein
MC: Mast cell
M‐CSF: Macrophage colony‐stimulating factor
MMP9: Matrix metallopeptidase 9
MO: Monocyte
MPO: Mieloperoxydase
NE: Neutrophil elastase
NET: Neutrophil extracellular trap
NGF: Nerve growth factor
NK: Natural killer
NLR: Neutrophil-lymphocyte ratio
PLA2G2A: Secretory phospholipase A_2_ group IIA
PMN: Polymorphonuclear cells
PTX3: Pentraxin 3
ROS: Reactive oxygen species
sPLA_2_: Secretory phospholipases A_2_
TCR: T cell receptor
TF: Tissue factor
TGF: Transforming growth factor
TLR: Toll-like receptor
TNF: Tumor necrosis factor
uPAR: Urokinase receptor
VEGF: Vascular endothelial growth factor
